# Looking Back on the First Anniversary

**Published:** 2013-01-01

**Authors:** Yogesh Kumar Sarin

**Affiliations:** Department of Pediatric Surgery, Maulana Azad Medical College, New Delhi.

The Journal of Neonatal Surgery (JNS) is now one year old [1]. As the first neonatal surgery journal anywhere in the world, we have done fairly well. We are now proud to present the first issue of the second volume- the anniversary issue. 

Last year the Journal received 88 manuscripts of which 61 were accepted for publication. The number of submissions is impressive for a fresh journal dedicated to the publication of high quality peer-reviewed papers. 

Many of authors are still very much attached to the tradition of seeing their papers as hard prints. Old habits are difficult to break, but the electronic print concept is gaining momentum globally. Some authors may also have reservations in publishing their articles in a new journal that is not yet included in PubMed. But we take pride in sharing that JNS is already indexed with many indexing agencies such as Directory of Open Access Journals (DOAJ), Indexcopernicus International, Google Scholar, NewJour, Open J-gate, Science Central, eDoctoronline, Ulrichsweb, and many more. 

We are committed to index the journal with Pubmed and Pubmed Central by next year. All the manuscripts submitted from the start would be available through Pubmed and Pubmed central.

JNS, unlike conventional hard copy journals, is not circulated in paper format. Hence, the editorial office is not under pressure to produce issues with a certain number of articles for mail delivery to subscribers. Only high quality articles with sound data have been accepted, a policy we intend to continue. The statistics regarding journal viewership are shown in illustration below (Fig. 1). A figure of more than 13000 viewers from around the world gives us a sense of accomplishment, though we aspire for improvement. The top five manuscripts that had highest viewership are listed below. We congratulate each of their authors. 

**Figure:**
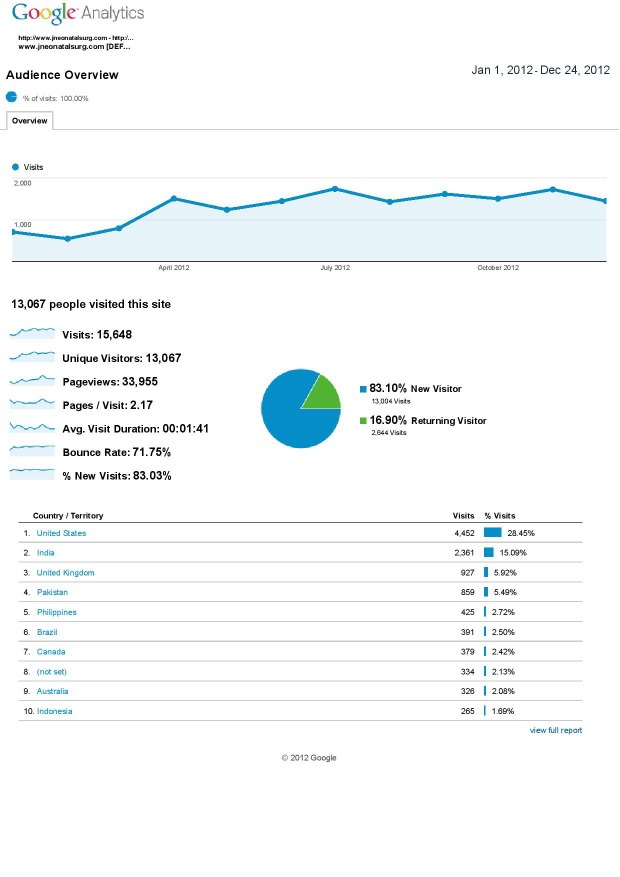
Figure 1: Journal's one year stats.

1. Raveenthiran V. Release of tongue-tie in neonates. J Neonat Surg 2012;1:15. (1820 views)
2. Reddy KR, Srinivas S, Kumar S, Reddy S, Hariprasad, Irfan GM. Sirenomelia a rare presentation. J Neonat Surg 2012;1:7. (1530 views)
3. Taneja B, Srivastava V, Saxena KN. Physiological and anaesthetic considerations for the preterm neonate undergoing surgery. J Neonat Surg 2012;1:14. (1322 views)
4. Akhtar T, Alladi A, Siddappa OS. Megacystis-microcolon-intestinal hypoperistalsis syndrome associated with prune belly syndrome: a case report. J Neonat Surg 2012; 1: 26. (1104 views)
5. Mitul AR, Ferdous KMN, Shahjahan M, Khan JG. Trans-fistula anorectoplasty (TFARP): our experience in the management of anorectovestibular fistula in neonates. J Neonat Surg 2012; 1: 36. (967 views)

I wish to express my sincere gratitude to our reviewers for their valuable time, as well as to our authors and the Journal’s Editorial Board for their most valuable support. The work of the reviewers and the editorial board is invaluable to the authors because it provides independent, objective, and detailed scientific evaluation necessary to impartially accept or decline a paper for publication. Thanks to their unbiased observations and their expanded comments, they have been able to choose the best manuscripts strictly based on quality, originality and significance.

We need to curb the menace of duplicate publication (self-plagiarism). I wish to have feedback from our authors and reviewers alike whether we should mandate our authors to declare if their work was submitted to and rejected by some other medical journal(s), although this shall have no bearing whatsoever on the acceptance of their work in our journal.

‘Athena’s pages’ and ‘Face the examiner’ have been our very popular sections and a recent suggestion to have the same topic for both the sections in a particular issue is welcome and worth a trial. We plan to add new sections such as Hypothesis, Art pertinent to neonatal surgery, and Resident corner where in trainees would be encouraged to write descriptive review of rare conditions and articles on neonatal surgical nursing.

I take the opportunity to thank our founder Editor-in-chief Prof. Afzal Shiekh who set the platform and put me into his shoes in October 2012. Please continue sending us your original research, reviews, views, and lending your valuable support.

## Footnotes

**Source of Support:** Nil

**Conflict of Interest:** The author belong to the editorial team, however the manuscript is dealt independently by other editors and the author did not participate in decision making of the manuscript.
